# Review of dietary patterns and gastric cancer risk: epidemiology and biological evidence

**DOI:** 10.3389/fonc.2024.1333623

**Published:** 2024-02-20

**Authors:** Ke Pu, Yang Feng, Qian Tang, Guodong Yang, Chuan Xu

**Affiliations:** ^1^ Sichuan Cancer Hospital & Institute, Sichuan Cancer Center, Affiliated Cancer Hospital of University of Electronic Science and Technology of China, Chengdu, Sichuan, China; ^2^ Department of Gastroenterology, Affiliated Hospital of North Sichuan Medical College, Nanchong, Sichuan, China; ^3^ Department of Neurosurgery, Xi’an No.3 Hospital, The Affiliated Hospital of Northwest University, Xi’an, Shaanxi, China; ^4^ Statesboro Office, Southeast Medical Group, Atlanta, GA, United States; ^5^ Department of Oncology & Cancer Institute, Sichuan Academy of Medical Sciences, Sichuan Provincial People’s Hospital, University of Electronic Science and Technology of China, Chengdu, Sichuan, China; ^6^ Department of Laboratory Medicine and Sichuan Provincial Key Laboratory for Human Disease Gene Study, Sichuan Provincial People’s Hospital, University of Electronic Science and Technology of China, Chengdu, Sichuan, China

**Keywords:** dietary patterns, gastric cancer, risk, priori and posteriori, review

## Abstract

Due to rapid research expansion on dietary factors and development of cancer prevention guidelines, the field of dietary pattern and its relationship to cancer risk has gained more focus. Numerous epidemiology studies have reported associations between Gastric Cancer (GC) and both data-driven posteriori dietary pattern and priori dietary pattern defined by predetermined dietary indexes. As dietary patterns have evolved, a series of patterns based on biological markers has advanced, offering deeper insights into the relationship between diet and the risk of cancer. Although researches on dietary patterns and cancer risk are booming, there is limited body of literature focusing specifically on GC. In this study, we compare the similarities and differences among the specific components of dietary patterns and indices, summarize current state of knowledge regarding dietary patterns related to GC and illustrate their potential mechanisms for GC prevention. In conclusion, we offer suggestions for future research based on the emerging themes within this rapidly evolving field.

## Introduction

Gastric Cancer (GC) remains a prevalent global cancer, ranking fifth in terms of incidence and fourth in terms of mortality worldwide. In 2020, there were approximately one million new cases and an estimated 700 000 deaths (1). With the effort of prevention measures, such as H. pylori prevalence reduction and food storage improvement, both incidence and mortality rate of GC have declined (2). Diet is a crucial modifiable lifestyle factor that plays a pivotal role in the development of gastric cancer. Various epidemiological studies have examined the association between the specific dietary factors, such as red meat, processed meat, white meat, vegetables and fruits, and beverage consumption, and the risk of gastric cancer, but the reported risk associated with individual dietary factors has shown inconsistency (3). Despite demonstrating an association between specific dietary factors and cancer risk, individual dietary constituents can have synergistic and antagonistic effects on disease risk. Conversely, assessing the dietary pattern as a comprehensive representation of one’s diet, obtained through self-reported questionnaires or dietary recalls, provides more robust effect estimates and results (4–6).

Traditionally, dietary patterns are mainly classified as posteriori dietary pattern and priori dietary pattern according to a set of predefined criteria (5). A posteriori dietary pattern is derived from cohort population data collected through self-reported food frequency questionnaire (FFQ) and is analyzed using statistical methods such as principle component analysis (PCA), factor analysis, or cluster analysis to identify dietary patterns (7, 8). *A priori* dietary patterns are developed based on existing knowledge about the relationships between food, nutrients, and disease (9). Several dietary score or index are used including country-specific guidelines, specific diets for chronic disease prevention and cultural eating habits (8). Country-specific guidelines encompass various indices such as the Health Eating Index (HEI), the Healthy Nordic Food Index (HNFI), and the Chinese Food Pagoda (CHFP), while diets for chronic disease prevention include the Alternative HEI and the Dietary Approaches to Stop Hypertension (DASH) diet. Cultural eating behaviors and traditions involve various dietary scores, such as Mediterranean Diet Scores (MDS), and vegetarian or vegan diet scores. Furthermore, several priori dietary indices have been developed to assess the effect of different dietary factors on cancer-specific biological processes or pathways, such as inflammation, insulin resistance, oxidative stress, and estrogen metabolism.

Previous reviews have delved into specific dietary patterns and various types of cancer, there is a shortage of comprehensive reviews that synthesize the literature on multiple dietary patterns and their relationship with gastric cancer risk. Additionally, the current understanding of how dietary patterns impact on gastric cancer incidence and mortality is limited. Therefore, our main emphasis is on GC cancer risk and prevention, we compare the similarities and differences among the specific components of dietary patterns and indices, and review their associations with gastric cancer risk. In conclusion, we provide suggestions for this rapidly expanding field and express our hope that tailored the dietary patterns for the prevention and treatment of gastric cancer will emerge in the future.

## A posteriori dietary patterns and gastric cancer

**Figure 1:**
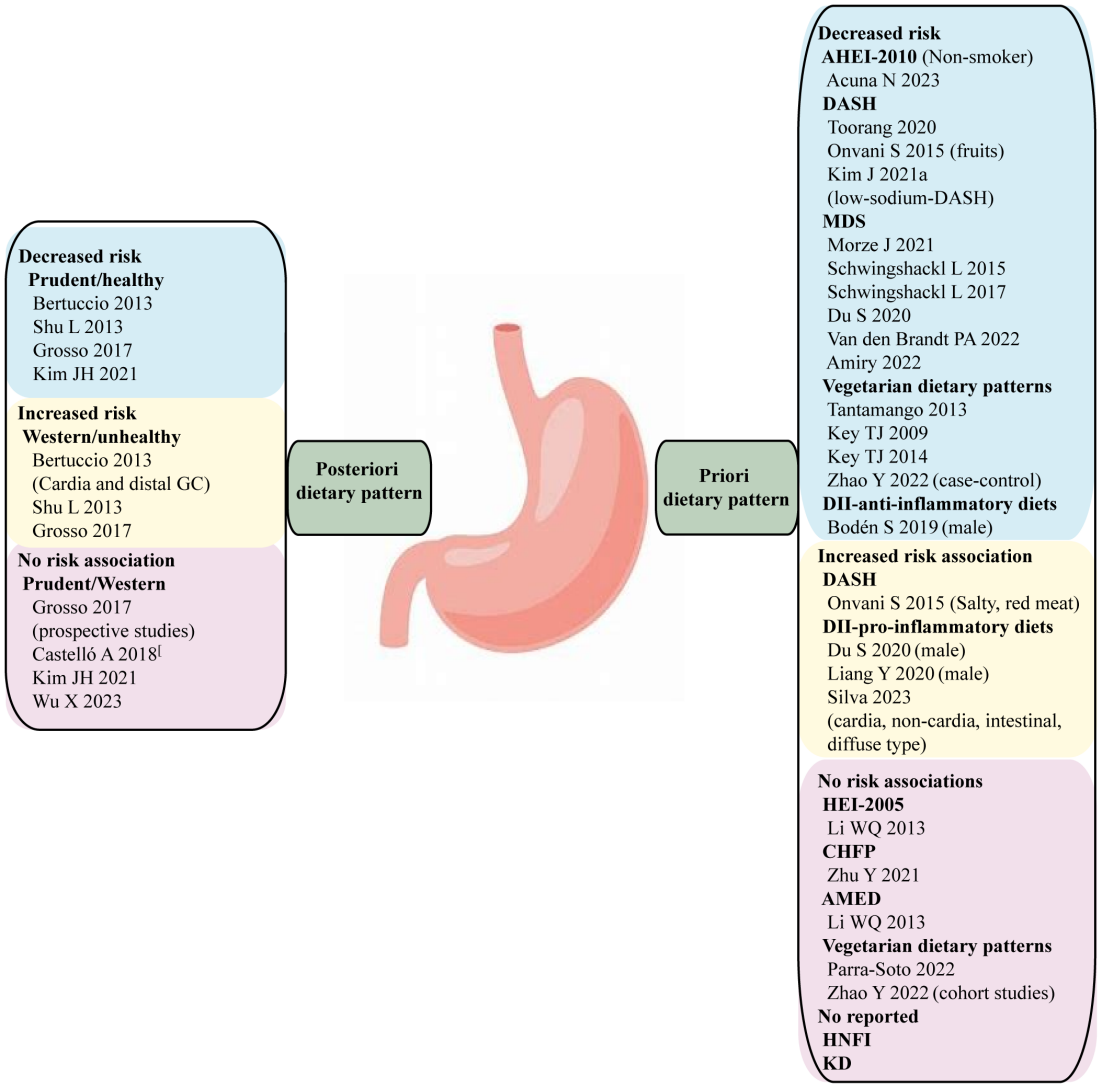
A posteriori and priori dietary patterns and cancer risk. The evidence is strongest for gastric cancer, where the alternative healthy eating index (AHEI-2010), Mediterranean diet score (MDS), and anti-inflammatory diets have been associated with reduced risk. There is no GC risk association with HEI-2005, Chinese food pagoda (CHFP), alternative mediterranean diets (AMED), and no consistent evidence for an association between the Dietary approaches to stop hypertension (DASH), vegetarian diets and gastric cancer.

Although the composition, weighting and labelling of dietary patterns can vary greatly across studies, the most commonly identified diets consist of unhealthy patterns, often labelled as “western” diets, and healthy patterns, often labelled as “prudent” diets. The western/unhealthy dietary pattern typically includes red and processed meats, sugary beverages, refined carbohydrates, and salty snacks. In contrast, the prudent/healthy diet is predominantly emphasized on vegetables and fruits. Several studies have examined the associations between posteriori dietary patterns and gastric cancer risk (**Figure 1**; **Supplementary Table 1**). A comprehensive systematic review and meta-analysis conducted by Bertuccio concluded that a high adherence to “prudent” diets was associated with a reduced risk of gastric cancer (OR 0.75, 95% CI: 0.63-0.90). Conversely, a high adherence to “western” diets was associated with an increasing risk of overall gastric cancer (OR 1.51, 95% CI: 1.21-1.89). Furthermore, the risk association between unhealthy dietary pattern and gastric cancer was more pronounced for cardia gastric cancer (OR 2.05, 95%CI: 1.51-2.78) than distal gastric cancer (OR 1.36, 95%CI: 1.07-1.73) (7). Another meta-analysis reviewed a total of 23 studies and found that individuals who followed a “healthy” dietary pattern had a significantly lower risk of gastric cancer (OR 0.69; 95% CI: 0.53-0.89). Conversely, adhering to an “unhealthy” dietary pattern was associated with a higher risk of stomach cancer (OR 1.59; 95% CI: 1.25-2.04) (10). In addition, a systematic review and meta-analysis conducted in 2017 included an additional twenty-one case-control studies (RR 1.41, 95% CI 1.15-1.72) and eight prospective cohort studies (RR 1.18, 95% CI 0.85-1.64) which supported the positive relationship between “western” diets and the risk of gastric cancer. In contrast, a meta-analysis of their thirteen case-control studies (RR 0.72, 95% CI 0.58-0.90) and eight prospective cohort studies (RR 0.98, 95% CI 0.74-1.29) of empirically derived dietary patterns indicated a reduced gastric cancer risk for individuals who followed “prudent” diets. However, prospective studies did not observe a significant association between dietary patterns and gastric cancer risk, regardless of whether individuals followed “prudent” and “western” diets (11).

Overall, these systematic reviews and meta-analyses consistently reported a reduced risk of gastric cancer with a “healthy” dietary pattern and an increased risk with an “unhealthy” dietary pattern in case-control studies. There are several other studies had inconsistent findings comparing to prior mentioned studies. A recent multi-case-control study in Spain (MCC-Spain) found no association between adherence to “western” diets and cardia tumor, meanwhile adherence to “prudent” diets showed no risk association with gastric cancer (12). Another case-control study conducted in the Korea population showed no clear association between adherence to “western” diets and gastric cancer, while “prudent” diets were associated with a lower risk of gastric cancer (OR 0.58, 95% CI 0.41-0.84) (13). Additionally, a case-control study conducted in China identified a positive risk association between “Fast food” pattern which is similar to “western” dietary pattern and gastric cancer. However, a protective effect was identified from the “Pickled food, processed meat products, and soy products” pattern within the context of unhealthy dietary pattern, Meanwhile, the “vegetable and fruit” pattern within the context of healthy dietary pattern showed no risk association with gastric cancer (14). The inconsistent finding regarding a posteriori dietary pattern in different centers can be attributed to many complex factors, including food sorts, recall bias of FFQ, total energy intake, and potential confounders. Large-scale prospective cohort study is suggested to provide further validation in the future.

## *A priori* dietary patterns and gastric cancer

Based on dietary guidelines and cultural eating practices, numerous *a priori* dietary patterns have been developed to assess their associations with different types of cancer. There is a lack of comprehensive reviews summarizing *a priori* dietary patterns and their relationship with gastric cancer risk.

## Healthy eating index and alternative healthy eating index

**Figure 2:**
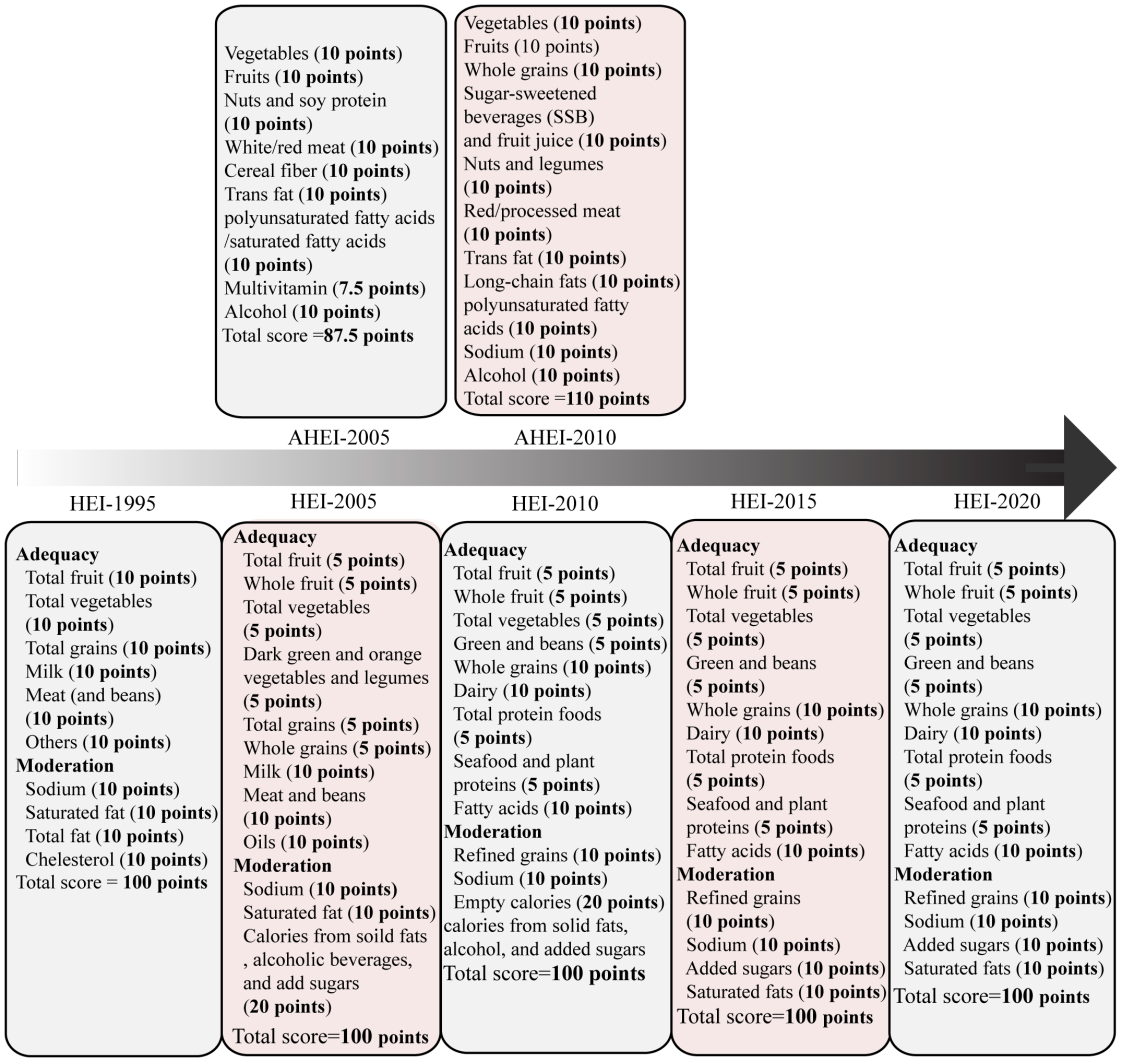
The Healthy Eating Index (HEI)-1995 has ten components, encompassing five food groups, four nutrients, and a measure of food intake variety (15). The HEI-2005 introduced changes from the previous version. On density basis of foods and nutrients, it adopted new food groups, such as whole fruit, dark green and orange vegetables, legumes, whole grains, and oils. Additionally, it included a discretionary component, which accounted for calories from solid fat, alcohol, and added sugar (SoFAAS) (16, 17). Building upon the HEI-2005, the HEI-2010 further refined the scoring system. It replaced the dark green and orange vegetables and legumes component with the greens and beans component, expanded the protein group by adding seafood and plant proteins, replaced oils and saturated fat with a fatty acids ratio, and introduced a moderation component by assessing over-consumption of refined grains instead of total grains (18). The HEI-2015 retained the components of the HEI-2010, except for replacing empty calories with saturated fat and added sugars. This modification resulted in a total of 13 components. Notably, excessive energy from alcohol was no longer accounted for in a separate component but was included within the total energy calculation. Legumes were also treated differently in the HEI-2015, as they were counted towards all four components instead of being allocated to either the vegetable or protein foods components (19). The HEI-2020 maintains the same 13 components and scoring standards as the HEI-2015 (20). Each component is assigned a score, with a maximum possible index score of 100. HEI scores > 80 indicate a “good” diet, scores ranging from 51 to 80 reflect a diet that “needs improvement,” and scores < 51 imply a “poor” diet (21). The AHEI score was based on the intake of nine individual components. The components include vegetables, fruits, nuts and soy, the ratio of white to red meat, cereal fiber, trans fat, the ratio of polyunsaturated fatty acids to saturated fatty acids, multivitamin use, and alcohol intake. Each component had the potential to contribute 0-10 points, intermediate intakes were scored proportionately to receive points between 0 and 10. Multivitamin use was dichotomous, either 2.5 points (did not use) or 7.5 points (did use). All individual component scores were then summed to obtain an AHEI score, which ranged from 2.5 to 87.5 (22). The AHEI-2010 comprises 11 components, six are associated with higher intakes being beneficial: vegetables, fruit, whole grains, nuts and legumes, long-chain ω-3 fatty acids, and polyunsaturated fatty acids. Moderate intake is recommended for alcohol. Conversely, four components should be limited or avoided: sugar-sweetened drinks and fruit juice, red and processed meat, trans fats, and sodium. To calculate the total AHEI-2010 score, each component is scored on a scale of 0 to 10. The total AHEI-2010 score is sum of each component score, ranging from 0 to 110, with a higher score indicating higher adherence to a healthy diet (23). The AHEI-2010 maintains most dietary components of AHEI but encourages people to consume long-chain n-3 fatty acids and reduce sugar intake.

In 1995, the Healthy Eating Index-1995 (HEI-1995) was developed by the U.S. Department of Agriculture (USDA) Center for Nutrition Policy and Promotion (CNPP) based on the Dietary Guidelines for Americans (DGA). This index aims to evaluate the dietary quality of individuals and populations. HEI-1995 is applicable for almost all age groups and comprises 10 components, including total fruits, total vegetables, total grains, dairy, meats, others, saturated fats, sodium, total fat, and cholesterol. The maximum score for HEI-1995 was 80 points, as shown in **Figure 2** and **Table 1** (15). The original HEI was initially updated in 2005 (HEI-2005) and has since been revised every 5 years in accordance with the dietary guidelines (16, 18–20). The last version, HEI-2020, comprises 13 components, including total fruits, whole fruits, total vegetables, greens and beans, whole grains, dairy, total protein, foods, seafood and plant proteins, fatty acids, refined grains, added sugars, saturated fats and sodium, the maximum score for HEI-2020 is 100 points. Scores exceeding 80 points indicates good dietary behavior (**Figure 2**, **Table 1**) (20). The Alternative Healthy Eating Index (AHEI) was developed in 2002 as a tool for chronic disease prevention, building upon the HEI. Based on prior observational studies, the AHEI initially included nine dietary items (22). In 2010, the AHEI was updated (AHEI-2010) to incorporate additional dietary factors associated with chronic disease (24). Both the HEI and AHEI can be utilized to assess diet quality, dietary patterns, and disease risk prediction (18). The alternative HEI incorporates several components from original HEI and introduces additional items such as alcohol consumption, *trans* fat, and sugar-sweetened beverages (**Figure 2**) (30), both the HEI and AHEI offer quantitative scoring for qualitative dietary guidance (**Table 1**) (22). These indices serve as valuable tools for evaluating the quality of diets, understanding dietary patterns, and assessing the potential risk of developing diseases.

**Table 1:** Components and scoring criteria of Health Eating Index, Alternative Health Eating Index, Dietary Approaches to Stop Hypertension, Mediterranean Diet Score, Alternate Mediterranean Diet, Health Nordic Food Index, and Chinese food pagoda.

Foods categories	Foods components	HEI-2005 (Guenther et al. (16)) (0-100ʹ)	HEI-2010 (Guenther et al. (18)) (0-100ʹ)	HEI-2015 (Krebs-Smith et al. (19)) (0-100ʹ)	HEI-2020 (Shams-White et al. (20)) (0-110ʹ)	AHEI (McCullough et al. (22)) (0-87.5ʹ)	AHEI-2010 (Chiuve et al. (24)) (0-110ʹ)	DASH (Fung et al. (25)) (0-8ʹ)	MDS (Trichopoulou et al. (26)) (0-9ʹ)	AMED (Fung et al. (27)) (0-9ʹ)	HFNI (Gunge et al. (28)) (0-6ʹ)	CHFP (Jiang et al. (29)) (0-45 ʹ)
Fruits and vegetables	Total fruits	≥ 0.8 c-eq/1,000 kcal (**5ʹ**)				NA		≥ 4.1 servings/d (**1ʹ**)	≥ median Fruits+nuts (**1ʹ**)	≥ median (**1ʹ**)	NA	>100 g/d (**5ʹ**)
	Whole fruits	≥ 0.4 c-eq/1,000 kcal (**5ʹ**)				≥ 4 servings/d (**10ʹ**)		NA	NA		≥ 56/71 g/d (M/F) Apples+ pears (**1ʹ**)	NA
	Total vegetables	≥ 1.1 c-eq/1,000 kcal (**5ʹ**)				≥ 5 servings/d (excluding potatoes) (**10ʹ**)		≥ 4.6 servings/d (excluding potatoes+ legumes) (**1ʹ**)	≥ median (**1ʹ**)	≥ median (excluding potatoes) (**1ʹ**)	≥14/16 g/d (M/F) cabbage (**1ʹ**) ≥16/29 g/day Root vegetables (**1ʹ**)	>400 g/d (**5ʹ**)
	Dark green and orange vegetables + legumes	≥ 0.4 c-eq/1,000 kcal (**5ʹ**)	NA			NA		NA	NA		NA	NA
	Greens and beans	NA	≥ 0.2 c-eq/1,000 kcal (**5ʹ**)			NA		NA	NA		NA	NA
Grains	Total grains	≥ 3.0 oz eq/1,000 kcal (**5ʹ**)	NA			NA		NA	NA		≥113/63 g/d (M/F) Rye bread (**1ʹ**)	>300 g/d (**5ʹ**)
	Whole grains	≥ 1.5 oz eq/1,000 kcal (**5ʹ**)	≥ 1.5 oz eq/1,000 kcal (**10ʹ**)			15 g/d (Cereal fiber) (**10ʹ**)	≥ 90 g/d (M), ≥ 75 g/d (F) (**10ʹ**)	≥ 2.4 servings/d (**1ʹ**)	≥ median Cereals (bread+potatoes) (**1ʹ**)	≥ median (**1ʹ**)	≥21/21g/d (M/F) Oatmeal (**1ʹ**)	NA
Protein foods	Dairy	≥ 1.3 c-eq/1,000 kcal (**10ʹ**)				NA		≥ 2.3 servings/d (Low-fat) (**1ʹ**)	< median Dairy products (**1ʹ**)	NA	NA	>300 g/d Dairy(**5ʹ**)
	Meat and beans	≥ 2.5 oz eq/1,000 kcal (**5ʹ**)	NA			NA		NA	NA		NA	>30 g/d (beans) (**5ʹ**)
	Total protein foods	NA	≥ 2.5 oz eq/1,000 kcal (**5ʹ**)			White/red meat = 4 (**10ʹ**)	0 servings/d (Red+processed meat) (**10ʹ**)	NA	NA		NA	NA
	Seafood and plant proteins	NA	≥ 0.8 oz eq/1,000 kcal (**5ʹ**)			≥ 1 serving/d (Nuts and soy)(**10ʹ**)	≥ 1 serving/d (Nuts and legumes) (**10ʹ**)	≥1.5 serving/d (Nuts and legumes) (**1ʹ**)	≥ median; Legumes (**1ʹ**) Fish (**1ʹ**)	≥ median; Legumes (**1ʹ**) Fish (**1ʹ**) Nuts (**1ʹ**)	≥ 42/35 g/d (M/F) Fish (**1ʹ**)	>50 g/d; Fish+shrimp (**3ʹ**)
Fats	Fatty acids	NA	PUFAs+MUFAs/SFAs ≥2.5 (**10ʹ**)			PUFAs/SFAs ≥ 1 (**10ʹ**)	EPA+DHA, 250mg/d PUFA ≥ 10% energy (**10ʹ**)	NA	MUFAs/SFAs ≥ median (**1ʹ**)	MUFAs/SFAs ≥ median (**1ʹ**)		
	Oils	≥12 g/1,000 kcal (**10ʹ**)	NA			NA		NA	NA		NA	
Moderation	Refined grains	NA	≤ 1.8oz.eq/1,000kcal (10ʹ)			NA		NA	NA		NA	NA
	Sodium	≤ 0.7g /1,000kcal (**10ʹ**)	≤ 1.1g/1,000kcal (10ʹ)			NA	lowest decile (**10ʹ**)	< 1.04g/d (**1ʹ**)	NA		NA	< 6g/d (**5ʹ**)
	Calories from SoFAA	≤ 20% energy (**20ʹ**)	≤19% energy (20ʹ)	NA		NA	NA	NA	NA		NA	NA
	Added sugars	NA	NA	≤ 6.5% energy (10ʹ)		NA	0 servings/d (SSB and fruit juice) (**10ʹ**)	0 servings /d (SSB) (**1ʹ**)	NA		NA	NA
	Saturated fat	≤ 7% energy (**10ʹ**)	NA	≤ 8% energy (10ʹ)		NA	0 servings/d (Red and processed meat) (**10ʹ**)	< 0.4 servings/d (Red and processed meat) (**1ʹ**)	< median (Meat and poulty) (**1ʹ**);	< median (Red and processed meat) (**1ʹ**)	NA	<30g/d Fats and oils (**5ʹ**) <50g/d Eggs (**3ʹ**) Meat + poultry < 100 g/d (**4ʹ**)
	Trans fat	NA				≤ 0.5% energy (**10ʹ**)	≤ 0.5% energy (**10ʹ**)	NA	NA		NA	NA
	Alcohol	NA				1.5-2.5 servings/d (M), 0.5-1.5 servings/d (F) (**10ʹ**)	0.5-2.0 servings/d (M), 0.5-1.5 servings/d (F) (**10ʹ**)	NA	10-50 g/d (M) 5-25 g/d (F) (**1ʹ**)	5-15 g/d (**1ʹ**)	NA	NA
	Multivitamin	NA				≥5 years **(7.5ʹ**)	NA	NA	NA		NA	NA

HEI, Healthy eating index; AHEI, Alternate healthy eating index; DASH, Dietary approaches to stop hypertension; MDS, Mediterranean diet score; AMED, Alternate Mediterranean diet; HFNI, Health Nordic food index; CHFP, Chinese food pagoda; Oz-eq, ounce equivalent; C-eq, Cup equivalent; EPA, Eicosapentaenoic acid; DHA, Docosahexaenoic acid; SSB, Sugar sweetened beverage; PUFAs, Polyunsaturated fatty acid; MUFAs, Monounsaturated fatty acid; SFAs, Saturated fatty acid; M, male; F, female; NA, not available; SoFAA, solid fats, alcohol and add sugars

Currently, there is limited study on the association between high scores on the HEI or AHEI and the risk of gastric cancer (**Figure 1**). A large cohort study conducted by National Institute of Health (NIH)-AARP Diet and Health study analyzed the relationship between HEI-2005 and the risk of gastric cardia adenocarcinoma (GCA) and gastric non-cardia adenocarcinoma (GNCA), but no direct association was found (31). A systematic review and meta-analysis examined the adherence to HEI (HEI, HEI-2005, HEI-2010) and AHEI (AHEI, AHEI-2010) dietary patterns, a high adherence to these patterns was associated with a reduced risk of total cancer-specific mortality (30). However, this study did not specifically investigate the association with gastric cancer. Another study conducted on a multiethnic cohort population found no significant associations between the AHEI-2010, HEI-2015 and gastric cancer for either anatomic site. However, in stratified analysis based on smoking status, it was observed that adherence to a high-quality AHEI-2010 diet among non-smokers appeared to decrease the risk of distal gastric adenocarcinoma (HR 0.60, 95% CI: 0.41, 0.88). It is important to note that the stratified result did not account for the heterogeneity of smoking status, making it difficult to explain (32).

Overall, the current evidence regarding the association between high scores on the HEI or AHEI and the risk of gastric cancer is limited. While some studies have explored the relationship between these dietary patterns and cancer-specific mortality, specific findings for gastric cancer are lacking. Further research is necessary to gain a better understanding of the potential impact of HEI and AHEI on the risk of developing gastric cancer.

## Dietary approaches to stop hypertension

DASH diet is initially developed as a dietary pattern to reduce hypertension. It emphasizes the consumption of fruits, vegetables, and low-fat dairy products, as well as diets with reduced saturated and total fats (**Table 1**). The effectiveness of DASH diet in lowing blood pressure has been validated through multi-center clinical trials (33). The traditional DASH diet score incorporates eight dietary categories: fruits, vegetables, nuts and legumes, whole grains, low-fat dairy products, sodium, red and processed meats, and sugary drinks. Each category is scored based on quintiles according to the intake of the corresponding dietary item, the total score ranges from eight to forty points, with higher scores indicating better adherence to the DASH diet (25).

The DASH diet is recognized as a healthy dietary guideline and has been associated with a reduced incidence of stroke, coronary heart disease, and metabolic syndrome (34). However, there has been limited research exploring the association between DASH diet and gastric cancer (**Figure 1**). A recent case-control study from Iran found that following the DASH diet was associated with a 54% reduced risk of gastric cancer (OR 0.46, 95% CI 0.26-0.83) (35). Furthermore, a review by Onvani et al. examined several meta-analysis studies and found that components of the DASH diet, such as high salty intake, red meat consumption, were positively associated with gastric cancer risk, while fruits were identified as a protective factor for gastric cancer to reduce incidence (36). Additionally, a Markov cohort state-transition model was employed to predict the impact of a low sodium-DASH diet on gastric cancer risk. The finding revealed that individuals who adhered to the low-sodium-DASH had a 24.8% lower risk of gastric cancer in male and 21.2% lower risk in female (37).

The mechanism underlying the negative correlation between adherence to the DASH diet and gastric cancer risk is not yet fully understood. However, it is hypothesized that the components of the DASH dietary pattern may influence the risk of developing gastric cancer. One potential explanation is the presence of lactic acid bacteria found in fermented dairy products in the DASH diet may inhibit the growth, invasion, and inflammation of *H. pylori* to reduce the incidence of gastric cancer (38). Additionally, low-sodium intake may help protect gastric mucosal cells from damage (39, 40). It can also prevent alternations in mucin production and the accumulation of chemical carcinogenesis induced by high levels of dietary salty (41). Furthermore, the protective effects of DASH diet against gastric cancer risk can be attributed to its low intake of red and processed meats as well as sugar-sweetened beverage (42). Red and processed meats have been linked to the production of carcinogens such as N-Nitroso compounds (NOCs), heterocyclic amines and polycyclic aromatic hydrocarbons during high-temperature cooking for a prolonged duration (43). Limiting the consumption of sweetness beverage can help lower insulin resistance, which played a pivotal role in the development of gastric cancer (44).

## Country-specific diet indexes

Country-specific dietary guidelines have not received as much extensive research attention as the HEI. However, some evidence suggests associations between these guidelines and cancer risk. The Health Nordic Food Index (HFNI), which was initially developed by Olsen to extract only foods with healthy effects of traditional Nordic Diet. The HFNI includes foods such as fish, cabbages, whole grain rye and oats, apples and pears, and root vegetables. Each food item consumed above the sex-specific median intake is assigned one point (**Table 1**) (28, 45). Thus far, research indicates that adherence to the HFNI may reduce the incidence of colon cancer (46), However, no studies have yet shown an association between HFNI and gastric cancer (**Figure 1**).

In 2007, the Chinese Nutrition Society and Ministry of Health released the Chinese dietary guidelines, known as Chinese food pagoda (CHFP). The CHFP 2016 score is based on five food groups and a sum of 12 specific foods, containing grains and cereals, vegetables and fruit, animal products (such as eggs, aquatic products, meats and poultry), soybeans and nuts, milk and its products, oil, and salt (**Table 1**) (29). Similar to the DASH diet, CHFP recommends the consumption of whole grains, vegetables, fruit, dairy products, and soy foods, moderate amounts of animal meats, and limited amounts of fat and salt (47). Limited research is available on whether adherence to the CHFP can reduce the risk of gastric cancer (**Figure 1**). In a recent study analyzing index-based dietary patterns and their association with gastric cancer in the Chinese population, researchers assessed adherence to CHFP-2016 using the modified Chinese Healthy Eating Index (mCHEI). The findings revealed no significant risk relationship between gastric cancer and mCHEI-2016. However, when adjusting for body mass index as a covariate, an inverse association was observed specifically among normal-weight subjects (48). Another study explored the potential of adherence to the CHFP in reducing overall cancer mortality, but no specific association with gastric cancer was identified (29).

## Mediterranean diet scores

The Mediterranean diet (MD) attracted attention from researchers in the 1960s when it was observed that mortality from cardiovascular disease in Italy, Greece and Spain was lower than that in northern Europe and the USA. Gradually, evidence has accumulated supporting the value of the MD for prevention of atherosclerosis, insulin resistance, metabolic syndrome, type 2 diabetes, obesity, and certain types of cancer (49). The MD is characterized by a high amount of extra virgin olive oil, vegetables, fruits, cereals, nuts, and legumes, moderate intakes of fish and other meat, dairy products, and red wine, and low intakes of eggs and sweets (**Table 1**) (26, 50). The traditional MD score (tMDS) includes nine components, each evaluated on a scale of zero to nine points. One point is given for the reference intake of components like vegetables, fruit and nuts, legumes, grains, fish, and monounsaturated fat/saturated fat. Conversely, one point is lost for consumption more than the median for dairy and meat. For alcohol, a value of one was assigned to a specific range (5-25 g/day for women and 10-50 g/day for men), alcohol is scored reversely when it is not consumed moderately. The alternate Mediterranean diet score (aMED) was proposed by Fung et al, to assess adherence to the MD. It modified the tMED by excluding potato products from the vegetable group, categorizing fruit and nuts separately, including whole grains instead of cereals, replacing “meats” with red and processed meat, canceling the dairy group, and assigning the same alcohol intake (5-15 g/d) for both sexes (27, 51).

Meta-analyses have shown that subjects who adhered closely to the MD have a decreased risk of gastric cancer (52–54). The recent updated systematic review and meta-analysis included a total of seven studies (three case-control and four cohort studies) from 2010 to 2019, this meta-analysis revealed adherence to MD was associated with a 30% reduction risk for gastric cancer (RR: 0.70, 95% CI 0.61, 0.80) (54). Subsequent studies have also observed higher consumption of MD may reduce gastric cancer risk (OR 0.42, 95% CI 0.2-0.86), but not necessary for prolonging the survival time of gastric cancer (HR 0.89, 95% CI 0.68-1.17) (55). Another study utilized the MD to develop a healthy lifestyle score (HLS) and found that for every 1-point increase in HLS, the HR decreased by 23% for GCA and 18% for GNCA, indicating HLS may significantly reduce the risk of gastric cancer (56). Additionally, a case-control study based on a Afghanistan hospital also revealed that individuals with the highest MD score had an 83% decreased risk of gastric cancer than those in the lowest tertile (OR 0.17, 95% CI: 0.03-0.80) (57). In contrast, Li et al. suggested aMED scores were not significantly associated with GCA or GNCA (31) (**Figure 1**).

The protective effect of MD against gastric cancer is not only attributed to the individual components but rather the synergy effect of the foods pattern (58). The anti-tumor effect may be depended on various factors, including lipid-lowing, anti-inflammatory, anti-oxidative stress, anti-aggregating, medication of cancer-promoting factors (such as hormones and growth factors), suppression of cancer-related nutrient pathways through modifications in amino acid content, and gut microbiota-mediated metabolic changes (59). Firstly, MD promotes the dietary fiber and vitamins levels of individuals by encouraging vegetable and fruits intake. Vitamin C, known for its ability to protect DNA from oxidant-mediated damage, may also protect the gastric mucosa against *H. pylori* colonization (60–62). Consumption of vegetable fiber can modify the metabolism of sex steroid hormones (63, 64). Numerous studies have found steroid hormones may lower the risk of gastric cancer, although the potential mechanism remains unclear (65, 66). Furthermore, various phytochemicals in whole grains and extra-virgin olive oil may be responsible for the anti-inflammatory and antioxidant effects of the MD (67). Among the phytochemical more important in plant foods are polyphenols, especially flavonoids account for nearly 60% of the known polyphenols (68). The efficacy of several flavonoids has been demonstrated in the prediction and treatment of gastric cancer (69, 70). Additionally, higher intake of omega-3 fatty acid appears to reduce circulating inflammatory markers and triglycerides (71). However, no significant association is observed between omega-3 fatty acid consumption and the incidence of gastric cancer (72). Lastly, the different amino acids proportion between “western” diets and MD modify the metabolic microenvironment. In MD, dietary methionine constitutes less than 40% of the diet and has prolonged animal lifespan and anti-tumor effect. Restricting methionine intake has been shown to lower the obesity-related hormonal such as insulin-like growth factor1 (IGF-1) and leptin, and up-regulate the adiponectin and fibroblast growth factor 21 (FGF21) (73). Meanwhile, a low content of branched-chain amino acids (BCAA) such as leucine, isoleucine, and valine, promotes insulin sensitivity, and induce β cell metabolic stress (74, 75).

**Figure 3:**
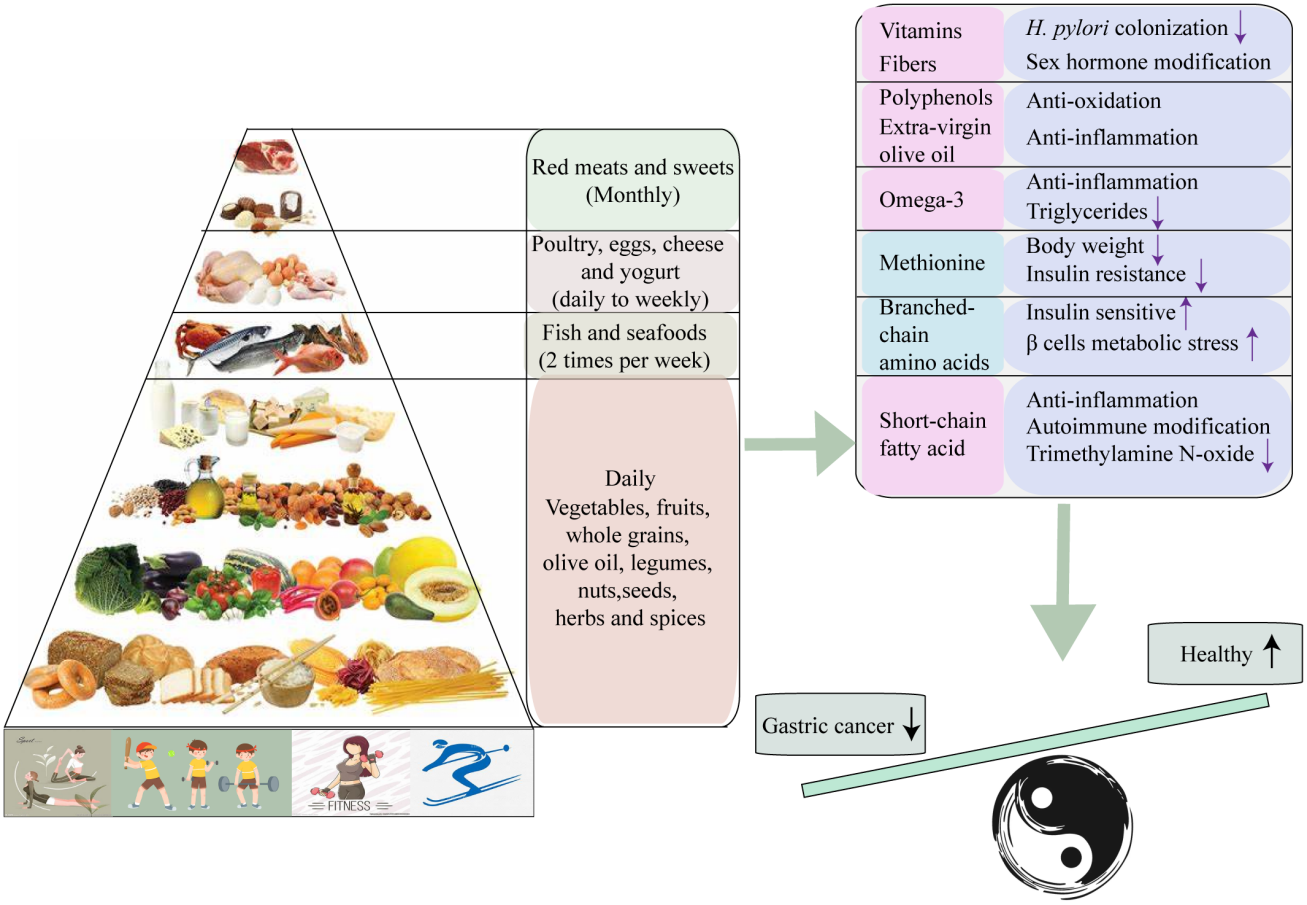
Collective mechanisms proposed to underlie the protective effect of Mediterranean diet against Gastric cancer.

The gut microbiota remodeling is another mechanism by which the MD exerts a protective role against gastric cancer. Long-term adherence to the MD can induce the alterations in the structure of gut microbiota community, specifically leading to an enrichment of Firmicutes and Bacteroidetes bacterial species. It can also increase gut short-chain fatty acid (SCFA) level, which have shown to suppress the development of several inflammatory, autoimmune, and allergic disease (76). Additionally, adhering to the MD can reduce urinary trimethylamine N-oxide (TMAO) that produced from dietary choline and L-carnitine (77, 78) (**Figure 3**).

## Vegetarian dietary patterns

Vegetarian diet is defined as a dietary profile characterized by the consumption of plant foods such as grains, legumes, nuts, seeds, vegetables, and fruit, while abstaining from meat and meat products, poultry, seafood and flesh from any other animal (79), there are two directions in vegetarianism: lacto-ovo-vegetarianism (LOV) and veganism (VEG). LOV includes dairy products, eggs, and honey together with plant-based foods. If eggs are excluded, it is referred to as lacto-vegetarianism (LV), while excluding dairy products is known as ovo-vegetarianism (OV). VEG mainly highlighted the consumption of purely plant-based foods (80). Vegetarian can be classified into other subgroups such as semi-vegetarian, pesco-vegetarian, and lacto-vegetarian. Semi-vegetarian is defined by infrequent consumption of red meat and poultry, typically occurring ≥ 1 time/month but <1 time/week. Pesco-vegetarian involves consuming fish ≥ 1 time/month, while limiting the intake of other meats to < 1 time/month. Lacto-vegetarian entails the consumption of eggs and dairy products ≥ 1 time/month, while fish and other meats are consumed <1 time/month, In contrast, vegans or strict vegetarians abstain from consuming eggs, dairy, and fish altogether.

Tantamango et al. conducted a comparative study on the cancer incidence of different vegetarian dietary patterns such as lacto, pesco, vegan, and semi-vegetarian, by comparison with non-vegetarians. The study included 2,939 cancer cases and found a significant association between vegetarian diet and cancers of the gastrointestinal tract (HR: 0.76; 95% CI: 0.63-0.90). Specifically, adherence to vegan diets took significant protection against overall cancer incidence (HR: 0.84, 95% CI, 0.72-0.99) and female-specific cancers (HR: 0.66, 95% CI, 0.47-0.92), while LOV diets appeared to be associated with a decreased risk of gastrointestinal system cancers (HR: 0.75; 95% CI: 0.60-0.92) (81). Key TJ et al. and his colleagues made the pooled analysis from five prospective studies to compare the mortality of gastric cancer between vegetarians and non-vegetarians. They found no significant differences in gastric cancer mortality between two groups (82). Subsequently, they continued to analyze the cancer incidence among vegetarians by pooling the individual participant data from the Oxford Vegetarian Study and EPIC-Oxford (83). Although long-term vegetarian diet management did show a reduced incidence of gastric cancer (RR: 0.36, 95% CI: 0.16-0.78), this pooled analysis exhibited a study heterogeneity, and the results should be interpreted with caution. In 2014, Key TJ et al. and his colleagues updated their analysis of cancer incidence in vegetarians using the data from the cohort population of Oxford Vegetarian Study and EPIC-Oxford. They amplified the sample size and extended the follow-up period, but the protective effect of vegetarian diets for gastric cancer still showed a significant heterogeneity (RR: 0.37, 95% CI: 0.19-0.69) (84). Inversely, a cohort study from UK biobank found that adherence to vegetarian diets did not influence gastric cancer risk comparing with meat-eaters (85). More recently, a meta-analysis based on a large population suggested plant-based diets reduced the risk of gastric cancer in case-control studies, but this protective effect was not observed in cohort studies (86) (**Figure 1**).

The unexplained anti-tumor effect of the vegetarian diet presents an intriguing area for further exploration. Vegetarians and vegans exhibit a more diverse gut microbiota compared to omnivores likely due to their consumption of carbohydrates and fibers. This diverse microbiota has the ability to synthesize some healthy compounds such as SCFA, phytochemicals and water-soluble vitamins, and plays a role in modifying lipid metabolism and lowering circulating levels of TMAO (87, 88). Furthermore, the restriction of dietary protein intake in vegetarian diets leads to the decreased levels of BCAA. This reduction in BCAA content can induce several metabolic effects, including enhanced fatty mobilization and improved insulin sensitivity (75, 89). Additionally, the limitation of methionine in vegetarian diets can contribute to a reduction in oxidative stress and inflammation within cells (90). These metabolic changes associated with vegetarian diets may potentially explain their anti-tumor effects.

## Patterns based on biological markers

Several dietary scores have been developed to evaluate the impact of various dietary factors on cancer-specific biological processes or pathways, specifically related to inflammation, insulin resistance, oxidative stress and estrogen metabolism. These scoring systems are categorized as *a priori* dietary patterns. Based on the literatures we reviewed regarding the association between gastric cancer and the dietary inflammatory index (DII) and ketogenic diet (KD).

## Dietary inflammatory potential

Foods, dietary factors, and non-nutritional components have the ability to influence both acute and chronic inflammatory responses. The DII score was initially proposed by Cavicchia et al. in 2007, drawing from the extensive literatures on diet and inflammation (91). It was further refined and updated using the dietary information from published studies during the period from 2007 to 2010 (92). The DII covers a sum of 45 food categories that are defined based on their content of anti-inflammatory and pro-inflammatory components. The DII reflects the dietary inflammatory potential status of individual’s diet. A higher score indicates a more pro-inflammatory dietary component, while a lower DII score suggests a more anti-inflammatory dietary component. During the development of DII score, IL-1β, IL-4, IL-6, IL-10, TNF-α and CRP were chosen to reflect the inflammatory effect of foods, each paper was assign a value based on the effect of the food on inflammation. If a food highly regulated the levels of IL-1β, IL-6, TNF-α and CRP, and simultaneously lowered the levels of IL-4 and IL-10, ‘+1’ was assigned the effect of pro-inflammatory. On the contrary, ‘-1’ was assigned the effect of anti-inflammatory. ‘0’ corresponded to the food did not involve in the change of inflammatory markers. Occasionally, these three variables may not explain certain situations where food parameters had both pro- and anti-inflammatory potential status. Shivappa and his team members developed more detailed scoring algorithm for DII. They introduced the two variables such as Z score and centered percentiles. Z score and centered percentiles were evaluated for each food parameter by using the world average intake and standard deviation. The centered percentile value for each food parameter multiplied the respective “overall food parameter-specific inflammatory effect score” to obtain the “food parameter-specific DII score”. Finally, all of the food parameter-specific DII scores were added to produce the overall DII score for an individual.

Currently, there are two studies have discussed the association between the DII and gastric cancer. Both of them found pro-inflammatory diets was associated with the increase incidence of gastric cancer (55, 93). Interestingly, the associations were only observed in males. Likewise, another cohort study found consistent and statistically significant associations between more anti-inflammatory diets and reduced risk of gastric cancer in men (HR 0.73, 95% CI 0.53-0.99) (94). A multicenter case-control study in Brazil displayed the relationship between DII and the anatomical region and histology types of gastric cancer. The study included energy-adjusted DII (E-DII) scores original from FFQ and found a pro-inflammatory diet was associated with a higher risk GA (OR 2.70, 95% CI 1.60-4.54), including GCA (OR 3.31, 95% CI 1.32-8.24), GNCA (OR 2.97, 95% CI 1.64-5.39), as well as both intestinal subtype (OR 2.82, 1.38-5.74) and diffuse subtype (OR 2.48, 1.23-5.00) (95) (**Figure 1**).

One of the possible mechanisms underlying the association between the DII and gastric cancer risk is the influence of diet-related chronic inflammation on the up-regulation of various cytokines and chemokines. This process can lead to the recruitment of numerous hematopoietic populations and progenitor cell populations to the inflamed gastric tissues (96). Inflammatory cytokines such as IL-1, IL-6, and TNF-α involve in the gastric cancer-related inflammation. IL-1 mainly generates from macrophage and has anti-tumor effect. Under the long-term exposure of chronic inflammation, it can activate some inflammatory factors with its subtypes IL-1a and IL-1β. Specifically, IL-1β can promote gastric cancer cell growth by the tyrosine kinase pathway (97). Meanwhile, it can also recruit MDSCs (myeloid-derived suppressor cells) by activating NF-κB pathway, which promotes the secretion of IL-6 and TNF-α to induce tumor proliferation. Additionally, higher level of IL-1β stimulates tumor angiogenesis to accelerate metastasis by activating vascular endothelial growth factors (VEGF) (98). IL-6 is associated with tumor advanced stages, invasion, and metastasis. It can regulate STAT3 phosphorylation that activates some transcription factors such as cFOX, TRF-1, and Bcl2, to enhance tumor cell growth, differentiation, angiogenesis, and adhesion. IL-6 also facilitates B-cell differentiation into plasma cells that enhances lymph node invasion and liver metastasis (99). TNF-α is known to be related to angiogenesis, progression, and metastasis in gastric cancer (100). TNF-α can induce carcinogenesis by activating NF-κB pathway (101). On the other hand, TNF-α promotes tumor development by upregulating the nitric oxide-dependent pathway and inhibiting DNA repair.

IL-4 is the major interleukin for T helper (Th)-2 mediated inflammation and is important for keeping Th1/Th2 balance (102). IL-4 has the potential to mediate alternatively activated macrophages (AAMs) polarization and inhibits pro-inflammatory cytokines (IL-1, IL-6, interferon-γ and TNF-α) secretion (102). Interesting, IL-4 has paradoxical roles in tumor immunity, it is necessary to know how one molecule has opposite effects (103). The immunosuppressive role of IL-10 has led to the general view that would facilitate tumor immune escape (104). Actually, IL-10 increases CD8 T cell infiltration and IFN-γ production, and favors effective T cell memory responses (105, 106), therefore, IL-10 may in gastric cancer be effective as an immunotherapy by potentiating the activity of antitumor CD8 T cells.

## Ketogenic diet

The inspiration of the ketogenic diet (KD) was from a presentation by Dr. Geyelin at the meeting of the American Medical Association, He described in detail the experience to use fasting for epilepsy (107). Concurrently, Dr. Wilder applied the concept of the KD to treat epilepsy again and deemed that KD could be thought as a longer-term alternative to fasting, Peterman then further developed the calculation of KD, which comprises a high-fat component (70-80%), very low carbohydrates (5%-10%), and adequate proteins (15-20%). The high proportion of fat led to produce acetone and beta-hydroxybutyric acid (β-HB), similar to the ketoemia induced by fasting (108, 109). Over the following decades, the beneficial effects of KD in neurological disorders, obesity, type 2 diabetes, cancer, intestinal disorders, and respiratory damage have attracted widespread attention (110). Adherence to KD can significantly lower blood triglyceride (TG) and cholesterol levels, and increase high-density lipoprotein (HDL) by cutting carbs (111), so does it enhance insulin sensitivity by decreasing serum insulin levels (112). Moreover, in the several days of the KD, the glucose source of glycogenesis transfers into glycerol from amino acid, at least 16% glucose generates from glycerol induced by TG-hydrolysis (113). Although the amount of glucoses is lower in the KD than those in complete fasting for several days (16% vs. 60%), the KD still decrease blood sugar levels and modifies the insulin/glucagon ratio to prevent energy deficiency induced by starvation (114, 115). Additionally, KD can take amounts of ketone bodies (KBs) due to the excessive production of acetyl coenzyme A (acetyl-CoA) and oxidation of fatty acids. Actually, the KBs as a more efficient energy source than glucose can bypass the glycolytic pathway to join the Krebs cycle directly (116), inhibit glycolysis and fatty acids, and activate fatty acid mediated peroxisome proliferator-activated receptor α (PPARα) (117).

KD has a widely application in the treatments of tumor and nervous disorder. Nevertheless, limited studies have been discussed the association between the KD and gastric cancer (**Figure 1**). Iyikesici et al. evaluated the therapeutic effects of the advanced gastric cancer patients on different treatments based on metabolically supported chemotherapy (MSCT) combined ketogenic diet, local hyperthermia and hyperbaric oxygen therapy (HBOT). During the mean follow-up time of 23.9 ± 12.7 months, the average overall survival was 39.5 months (95%CI: 28.1-51.0) and the mean progression-free survival was 36.5 months (95%CI: 25.7-47.2), thus, they thought these combination treatment at least appears to be promising for advanced gastric cancer patients (118). Another study found that the KD significantly improved the efficacy of Oldenlandia diffusa extract and curcumin in treatment of gastric cancer by increasing miR-340 expression and apoptosis mediated by autophagy, oxidative stress, and angiogenesis (119).

**Figure 4:**
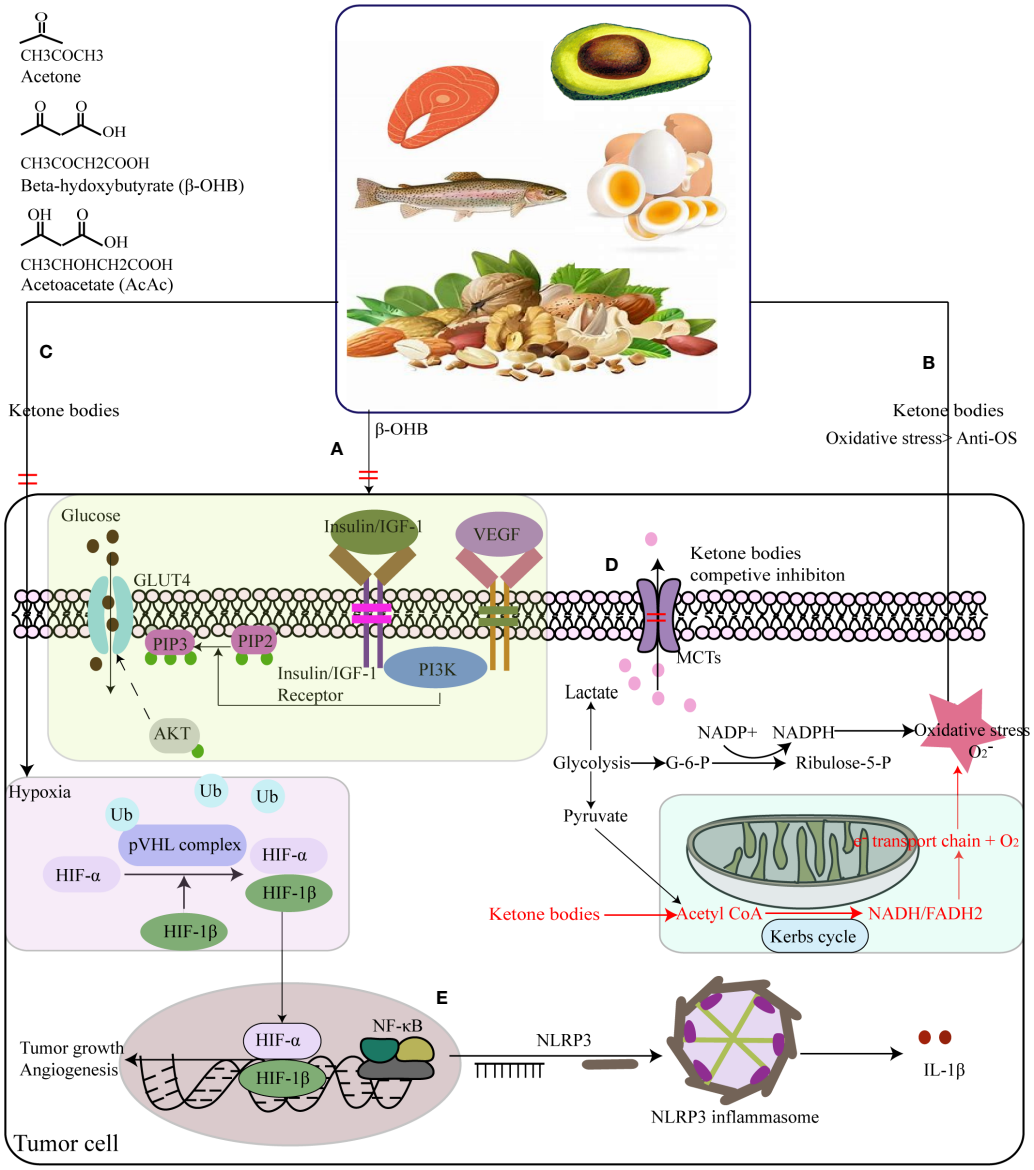
Summary of the potential interplay in the molecular mechanisms of the ketogenic diet (KD) and cancers. **(A)** KD lower insulin and IGF-1 levels to suppress tumor cells’ insulin-stimulated GLUT4 trafficking by PI3K/Akt signing pathway. **(B)** Mitochondrial DNA dysfunctions triggering into reactive oxygen species (ROS) overdosage in tumor cells, resulting in NADPH and pyruvate increase via the pentose phosphate shunt and glycolysis way individually to reduce hydroperoxides to keep the steady state of oxidative stress. **(C)** KD can reduce NADPH generation to increase the oxidative stress in tumor cells, by impeding gluconeogenesis to form glucose-6-phosphate (G-6-P) that is necessity for the pentose phosphate shunt. **(C)** KD treated ad libitum could down-regulated hypoxia-related protein. **(D)** Ketones enter into the cancer cell by the monocarboxylate transporters (MCTs) that is responsible for lactate export, leading to competitive inhibition of lactate export. **(E)** KD had anti-tumor effects by hindering systematic inflammation. Ketone bodies supplements inhibited the activation of NLRP3 inflammasome mediated by inhibiting NF-κB and STAT3 activation, and finally lower the expression levels of IL-1β.

The KD is recognized as a potential anti-cancer therapy due to its one of important abilities to modify glucose metabolism and reduce insulin signaling and IGF-1 (120–123). As result of Warburg effect, glucose from dietary carbohydrates provide a primary metabolic energy for many cancers. Therefore, gradually the KD is thought to be an anti-tumor therapy via dietary carbohydrate limitation. Although hyperglycemia is known risk factor to promote tumor growth (124, 125), KD indeed inhibit glucose uptake in cancer cells by lowering their glucose availability (126). Moreover, low levels of insulin and IGF-1 suppress the activation of PI3K/Akt/GLUT4 signaling pathway, reducing the glucose uptake and downregulating the membrane translocation of glucose transporters (127–129) (**Figure 4A**). Additionally, it also inhibits the insulin-related down-stream signaling molecules such as NF-κB, and vascular endothelial growth factor (VEGF) to exert a pro-apoptosis and anti-angiogenesis for cancers (130, 131). Apart from the effect of the KD on glucose metabolism and insulin concentration, it still influences the oxidative stress of cancer cells by inhibiting ROS production and enhancing endogenous antioxidant expression in cancers *in vivo* (132) (**Figure 4B**). In contrast, KD can cause shift in energy production pathways which potentially results in an elevation of oxidative stress within tumor cells. Normally, tumor cells generate NADPH through the pentose phosphate shunt and pyruvate via glycolysis, thereby reducing hydroperoxides. However, the fat metabolism with KD lacks the ability to undergo gluconeogenesis to produce glucose-6-phosphate (G-6-P), which is necessary for entering the pentose phosphate shunt and generating NADPH. Therefore, with KD, tumor cells are compelled to rely on mitochondrial metabolism for energy, ultimately impeding NADPH regeneration and increasing oxidative stress (133) (**Figure 4B**). Secondary, Woolf et al. found that *ad libitum* KD treatment down-regulated hypoxia-related protein (hypoxia-inducible factor 1, HIF-1α) and growth-driven proteins (NF-κB, and VEGF receptor-2), when assessing the effect of the KD on cancers growth and progression using a mouse model (134) (**Figure 4C**). Ketones enter into the cancer cell via the monocarboxylate transporters (MCTs) that is responsible for lactate export, leading to competitively inhibit lactate export and shorten cancer survival time (135) (**Figure 4D**). Finally, some studies have reported the KD exerts anti-tumor effects by hindering systematic inflammation mediated by NLRP3 inflammasome (136, 137), with the consequent reduction of inflammatory markers in cancers (124, 138, 139) (**Figure 4E**).

## Conclusions

Together with the burgeoning epidemiological studies of dietary patterns and cancer, many novel dietary patterns have been rapidly developed and introduced, however, achieving homogeneity across different studies investigating GC-related dietary patterns has proven challenging. Paradoxical finding frequently arise, throwing the need for large-scale cohort studies to validate these patterns and preventing uncertainty in guidelines development. It seemed an enigma to seek for specific dietary pattern to provide an effective prevention strategy for gastric cancer risk because the biological mechanisms linking different dietary patterns to gastric cancer risk are likely to involve synergistic or additive biological effects of the individual dietary components, and trigger change of the metabolites, gut microbiota, inflammation, and immune state. Meanwhile, studies on biological mechanisms predominantly rely on animal models, which may not provide an accurate representation for human subjects. Additionally, the customization of dietary recommendations based on susceptibility factors within sub-populations remains unknown and requires further development.

## Author contributions

KP: Conceptualization, Writing – original draft. YF: Writing – original draft. QT: Writing – review & editing. GY: Writing – review & editing. CX: Writing – original draft, Writing – review & editing.
