# Supplementary material for: Review of dietary patterns and gastric cancer risk: epidemiology and biological evidence
Source: Front Oncol. 2024 Feb 20;14:1333623. doi: 10.3389/fonc.2024.1333623 (PMC10912593; doi:10.3389/fonc.2024.1333623)
Supplement: Supplementary file 1 [file Table_1.docx]

Supplementary Material

## Supplementary Table

**Supplementary Table 1.** Summary of previous studies on posteriori dietary patterns and gastric cancer

| Reference | Study type | Country | No.of GC | No. of subject/ control | Age (years) | Dietary patterns method | Main results |
| --- | --- | --- | --- | --- | --- | --- | --- |
| Masaki 2003 | Cohort study  (JACC) | Japan | 84 | 5,644 | 40-69 | PCA | **No association:**  “Vegetable and fruit” pattern (**“prudent/healthy”**); “Western breakfast” pattern; “Meat” pattern (**“western/unhealthy”**); “Rice/snack” pattern |
| Kim 2004 | JPHC study Cohort 1 | Japan | 400 | 42,112 | 40-59 | PCFA | **Increase risk:**  “Traditional” pattern  **Decrease risk:**  “Healthy” pattern for female  **No association:**  “Healthy” pattern for male; “Western” pattern |
| Palli 2001 | Case-control | Italy | 382 | 561 | NA | PCFA | **Increase risk:**  “Traditional” pattern  **Decrease risk:**  “Vitamin-rich” pattern (**“prudent/healthy”**)  **No association:**  “Refined” pattern (**“western/unhealthy”**); “Fat-rich” pattern |
| Chen 2002 | Case-control | USA | 124  (EGA) | 449 | >=21 | CA | **No association:**  “Healthy” pattern; “High meat” pattern; “High salty snacks” pattern; “High desserts” pattern; “High milk” pattern; “High white bread” pattern |
| De Stefani 2004 | Case-control | Uruguay | 240 | 960 | 30-89 | PCFA | **Increase risk:**  “Starchy” pattern  **Decrease risk:**  “Healthy” pattern; “Mixed” pattern |
| Bahmanyar 2006 | Case-control | Sweden | 258 | 815 | <80 | PCFA | **Increase risk:**  “Western” pattern  **No association:**  “Healthy” pattern; “Alcohol drinker” pattern |
| Campbell 2008 | Case-control | Canada | 1169 GC  132 GCA  161 GNCA | 2332  886(subsample) | 20-74 | FA | **“Western” pattern:**  Increase total GC risk for both sexes, GCA for male, and for GNCA among female; No risk association of GCA with female, and GNCA with male  **“Prudent” pattern:**  Decrease total GC risk for female; No risk association of GCA and GNCA with sex |
| Bertuccio 2009 | Case-control | Italy | 230 | 547 | 22-80 | PCFA | **Increase risk:**  “Animal products” pattern (**“western/unhealthy”**); “Starch-rich” pattern  **Decrease risk:**  “Vitamins and fiber” pattern (**“prudent/healthy”**)  **No association:**  “Vegeble unsaturated fatty acids” pattern |
| De Stefani 2009 | Case-control | Uruguay | 274 | 2532 | NA | PCFA | **Increase risk:**  “Traditional” pattern; “Starch-rich” pattern.  **Decrease risk:**  “Prudent” pattern  **No association:**  “Western” pattern; “Drinker” pattern |
| Bastos 2010 | Case-control | Portugal | 591 GC  72 GCA  444 GNCA | 1463 | 18-93 | CA | **Increase risk:**  Total GC and GNCA (pattern II vs pattern I);  **No association:**  Pattern III vs pattern I. |
| Navarro Silvera.  2011 | Case-control | USA | 255 GCA  352 GNCA | 687 | 30-79 | PCFA | **Increase risk:**  “Meat/nitrite” pattern (**“western/unhealthy”**) for GNCA;  **Decrease risk:**  “Fruit/vegetable” pattern (**“prudent/healthy”**)  **No association:**  “Meat/nitrite” pattern (**“western/unhealthy”**) for CGA; “smoking/alcohol” pattern; “legume/meat alternate” pattern; “GERD/BMI” pattern; “fish/vitamin C” pattern (**“prudent/healthy”**) |
| Pham TM 2010 | Japan Collaborative Cohort study (JACC) | Japan | 477 | 63403 | 50-60 | PCA | **Decrease risk:**  “Dairy product” pattern for males  **No association:**  “Vegetable” pattern (**“prudent/healthy”**); “animal food” pattern (**“western/unhealthy”**); “dairy product” pattern for females |
| Denova-Gutierrez E 2014 | Case-control | Mexico | 248 | 478 | 59±8.0/60±10 | FA | **Increase risk:**  Pattern 3 for GC risk including negative *H. pylori*, positive *H. pylori*, intestinal and diffuse type;  **Decrease risk:**  Pattern 1 (**“prudent/healthy”**) for negative *H. pylori*-GC, and for intestinal GC;  **No association:**  Pattern 2 |
| Adela Castello 2018 | MCC-Spain study | Spain | 264 | 3040 | 63.9±11.4/65.9±126 | FA | **Increase risk:**  “Western” dietary pattern for GNCA and intestinal type  **No association:**  “Prudent” dietary pattern |
| Kim JH 2021 | Case-control | South Korea | 415 | 830 | 53.8±9.3/53.7±9.0 | PCFA | **Decrease risk:**  “Prudent” dietary pattern for positive *H. pylori*-GC  **No association:**  “Western” dietary pattern |
| Wu X 2023 | Case-control | Huaihe River Basin, China | 696 | 1772 | 65.4±9.6/63.3± 10.1 | PCA | **Increase risk:**  “Fast-food” pattern  **Decrease risk:**  “Flavor, garlic, and protein” pattern; “vegetable and fruit” pattern; “pickled food, processed meat products, and soy products” pattern; “non-staple food” pattern; “coffee and dairy” pattern. |

Abbreviation: JACC, Japan Collaborative Cohort study for Evaluation of Cancer Risk; JPHC, Japan Public Health Center-based prospective study; MCC, Multi-Case Control PCA, Principal Component Analysis; PCFA, Principal Component Factor Analysis; CA, Cluster Analysis; FA, Factor Analysis; GCA, Gastric Cardia Adenocarcinoma; GNCA, Non-cardia Gastric Adenocarcinoma; NA, Not Availability

**Reference**

Masaki, M., Sugimori, H., Nakamura, K., Tadera, M. (2003). Dietary patterns and stomach cancer among middle-aged male workers in Tokyo. *Asian Pac J Cancer Prev*. 4, 61-66.

Kim, M.K., Sasaki, S., Sasazuki, S., Tsugane, S., Japan Public Health Center-based Prospective Study Group. (2004). Prospective study of three major dietary patterns and risk of gastric cancer in Japan. *Int J Cancer*. 110, 435-442.

Palli, D., Russo, A., Decarli, A. Dietary patterns, nutrient intake and gastric cancer in a high-risk area of Italy. (2001). *Cancer Causes Control*. 12, 163-172.

Chen, H., Ward, M.H., Graubard, B.I., et al. Dietary patterns and adenocarcinoma of the esophagus and distal stomach. (2002). *Am J Clin Nutr*. 75, 137-144.

De Stefani, E., Correa, P., Boffetta, P., et al. Dietary patterns and risk of gastric cancer: a case-control study in Uruguay. (2004). *Gastric Cancer*. 7, 211-220.

Bahmanyar, S., Ye, W. (2006). Dietary patterns and risk of squamous-cell carcinoma and adenocarcinoma of the esophagus and adenocarcinoma of the gastric cardia: a population-based case-control study in Sweden. *Nutr Cancer*.54, 171-178.

Campbell, P.T., Sloan, M., Kreiger, N. (2008). Dietary patterns and risk of incident gastric adenocarcinoma. *Am J Epidemiol*. 167, 295-304.

Bertuccio, P., Edefonti, V., Bravi, F., et al. (2009). Nutrient dietary patterns and gastric cancer risk in Italy. *Cancer Epidemiol Biomarkers Prev*. 18, 2882-2886.

De Stefani, E., Deneo-Pellegrini, H., Boffetta, P., et al. (2009). Dietary patterns and risk of cancer: a factor analysis in Uruguay. *Int J Cancer*. 124, 1391-1397.

Bastos, J., Lunet, N., Peleteiro, B., et al. (2010). Dietary patterns and gastric cancer in a Portuguese urban population. *Int J Cancer*. 127, 433-441.

Navarro Silvera, S.A., Mayne, S.T., Risch, H.A., et al. (2011). Principal component analysis of dietary and lifestyle patterns in relation to risk of subtypes of esophageal and gastric cancer. *Ann Epidemiol*. 21, 543-550.

Pham, T.M., Fujino, Y., Kikuchi, S., et al. (2010). Dietary patterns and risk of stomach cancer mortality: the Japan collaborative cohort study. *Ann Epidemiol*. 20, 356-363.

Denova-Gutiérrez, E., Hernández-Ramírez, R.U., López-Carrillo, L. (2014). Dietary patterns and gastric cancer risk in Mexico. *Nutr Cancer*. 66, 369-376.

Castelló, A., Fernández de Larrea, N., Martín, V., et al. (2018). High adherence to the Western, Prudent, and Mediterranean dietary patterns and risk of gastric adenocarcinoma: MCC-Spain study. *Gastric Cancer*. 21, 372-382.

Kim, J.H., Lee, J., Choi, I.J., et al. (2021). Dietary patterns and gastric cancer risk in a Korean population: a case-control study. *Eur J Nutr*. 60, 389-397.

Wu, X., Zhang, Q., Guo, H., et al. (2023). Dietary patterns and risk for gastric cancer: A case-control study in residents of the Huaihe River Basin, China. *Front Nutr*. 10, 1118113.
